# Proceedings of First International meeting of Research Journal Editors organized by Islamic World Science Citation Center at Shiraz, Iran (October 23-24^th^ 2012)

**DOI:** 10.12669/pjms.291.3181

**Published:** 2013

**Authors:** Shaukat Ali Jawaid

Shiraz (Islamic Republic of Iran): Islamic World Science Citation Center in Shiraz in collaboration with ISESCO hosted the First International meeting of Research Journal Editors at ISC campus on October 23-24^th^ 2012. It was attended by a select gathering of Research Journal Editors in different subjects from many Islamic countries including Malaysia, Iraq, Egypt, Pakistan, and Bangladesh besides Islamic Republic of Iran. Research Journal Editors from Medical Sciences, Science and Technology, Economics, Agriculture, Environment, library sciences etc., were invited to share their knowledge and experience and introduce them to the functioning of Islamic World Science Citation (ISC) to strengthen this research database representing the Islamic Countries. 

**Figure:**
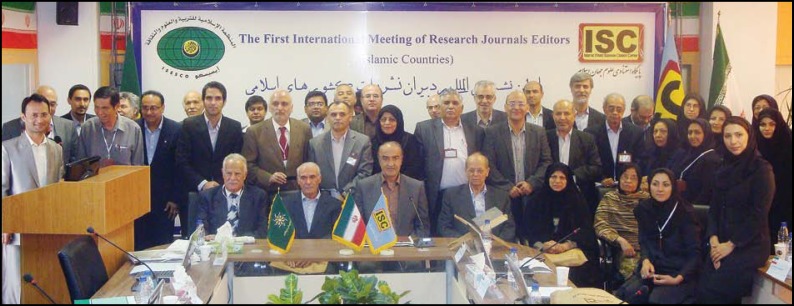
Group photograph shows some of the delegates photographed during the First International meeting of Research Journal Editors organized by ICS at Shiraz from October 23-24th 2012.

## Inaugural Session

 Speaking in the inaugural session, **Dr. Abbas Sadri** Director of ISESCO Regional Office referred to the impact of such academic activities has on improvement of science and technology as well as research. We collect information and provide it to ISESCO which then passes it on to the whole Islamic world which is the purpose of establishing this ISC. Islamic countries, he further stated, have their own values, problems and they need one body to evaluate performance of these countries in the scientific area. This conference will provide an opportunity to the experts to have face to face discussions in the related areas. ISC is still in infancy, it needs lot of improvement. There are obstacles and challenges in enhancement of quality of research and we hope to make progress in the days to come, he added.

**Prof. Abbas Hoori** Faculty member from Tehran University spoke about Scientific Journals and Evaluation Tools. Delay in publication for several months after the manuscript has been written makes print journals not an ideal medium. On the other hand online electronic publishing enables to publish immediately. By 2006 most of the journals have gone online and most libraries now buy electronic version of journals. The journals print only a few copies. Many universities purchase scientific license for electronic publications.

 Speaking about peer review, he said this is required to ensure quality of the manuscript before it is accepted for publication. Peer review is currently under discussion at different forums. Authors have to wait for scrutiny by peers. Peer review helps the authors to further improve the quality of their manuscripts. An article, he said, may be rejected because of poor quality if the reviewer’s comments are not addressed in the revised version. Peer Review ensures that articles and journals maintain high quality and standard and it also gives credibility. However, peer review may be used and abused. Post publication commentary can be helpful. 

 Talking about evaluation tools, he referred to Impact Factor (IF), Fignefactor, SCI Mago Rank, Expert Survey, Publication Power Approach (PPA) and the H. Index. There is prestige associated with these evaluation tools. Impact Factor is a major factor in citations. It is used for journal ranking. High Impact Factor is considered more important. There are some problems associated with Impact Factor to measure quality and validity of Impact Factor as a measure of journal evaluation. Some editors try to improve their Impact Factor by publishing more review articles which are cited more than original articles and case reports. Some editors with the help of reviewers force the authors to cite articles published in their own journals to improve their Impact Factor. Secondly in calculating Impact Factor the number of articles published by a journal affects their IF. He was of the view that weighted IF should be given more importance than IF alone. He then referred to some misuses of IF in evaluation procedures. He was of the view that it is more important to assess the quality of an individual article rather than a journal in which they are published. Some articles are never cited. It is ridiculous to judge the quality of journals based on one article. In SCI Mago Rank (SJR) takes into account the citation and prestige of the journal where they are cited. Citation coming from more prestigious journals is much more important. He also briefly discussed the quality of research in social sciences.

 He concluded his presentation by highlighting the importance of new ideas in scholarly approach which these journals present and validity of journals. Techniques of evaluation of journals have its own strengths and weaknesses. Evaluation tools, he opined, are tools and it matters a lot how you use them. A technique may be helpful at one place and harmful at other place, he added.

**Prof. Jafar Mehrad** President of ISC said that ISC is a multilingual full text system which is different from the other two well known databases i.e. Thompson-Reuter and Scopus as they only cover English language journals. Both these systems are highly reputed and index many international authoritative journals. Scopus covers about twenty thousand while Thomson Reuters covers about ten thousand research journals. Though these systems have different approaches regarding citation analysis both have significant rules in evaluating the scientific performance of the countries. Recently these two systems have also included journals published in languages other than English. They have abstracting and indexing services. ISC does not have more than five years of experience. It is fully supported by Islamic countries which is utmost important for its success.ISC covers all the research journals published in English, Persian as well as Arabic. We generate Journal Citation Reports in all these languages and plan to have citation analysis in other languages like Turkish, Malay and French as well in the days to come. More recently countries like Germany, England, India and Poland have expressed their interest to get their journals indexed in ISC. Hence entrance of these journals of non-Islamic countries to ISC will enable it to go beyond the borders of Islamic World and consequently become an international indexing system. ISC will enable scientific mapping of Islamic countries. ISC, he further stated is approved by the Ministers of Science and Technology of the Islamic countries and we in Iran are proud to be hosting it as per unanimous decision of the Organization of Islamic Countries (OIC).

 ISC, Dr. Jafar Mehrad said is currently pursuing its expansion and growth in several areas. We had been studying and analyzing the scientific Persian journals since 1999 in different ISC systems and products. Analysis and indexing of Arabic Research Journals was stated in 2005 and it was followed by evaluation and analysis of English Language Journals from Iran and other Islamic countries. Indexing and processing of Turkish, Malay, French and other national languages is being planned along with development of ISC’s organization. ISC, he said incorporates a number of sub-systems each targeting a specific objective. With the availability of ISC’s services at their disposal the Organization of Islamic Countries might utilize these services to instigate research-scientific cooperation within and between the Islamic countries. ISC enables scientific mapping of OIC universities with the objective to clarify the current research scientific status of each member state. This kind of scientific mappings is a great help in production of map of science in the Islamic World.

 Referring to the scientific research output in Iran, Dr. Jafar said that in 1990 the number of research papers published were one hundred ninety which increased to 26,740 in the Year 2011. Similarly there has been tremendous improvement in many Islamic countries like Turkey, Malaysia, and Kingdom of Saudi Arabia etc. ISC, Dr. Jafar further stated has been ranking the Iranian universities and research institutions during the past two years. The criteria and indicators defined for the ranking system was approved in the Islamic Conference of Ministers of Higher Education and Scientific Research held in Riyadh Saudi Arabia on October 4-5^th^ 2011.

 Giving details of the ISC set up, Dr.Jafar said that it has a steering council, an Executive Committee and Scientific Council. Its website (www.isc.gov.ir) can be accessed to have all the details. He urged all the participants to help disseminate this information about ISC and strengthen this database which will be extremely useful for academicians and researchers. ISC covers 237 medical journals from Iran. We are proud of where we are and what we are doing, he added.

**Figure:**
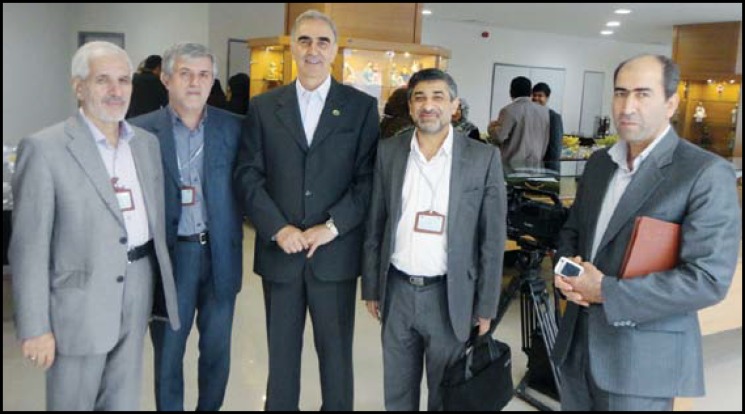
Prof. Jafar Mehrad President of ICS photographed along with some of the delegates to the ICS conference for Research Journal Editors held at Shiraz from October-23-24, 2012.

## Peer Review Seminar

**Prof. Rahmatoolah Fattahi** from Mashad Ferdowsi University, Mashad talked about Peer Review issues assuring the quality of knowledge for the 21^st^ century. He discussed in detail the problems and challenges in peer review and how to improve it. Scientific papers, he felt, must have some requirements to maintain quality of knowledge. It also depends on the quality of reviewer’s judgment. Scholarly papers are linked with good peer review, quality criteria’s and research.There is a huge body of knowledge, the existing knowledge, new knowledge which goes through peer review for quality assurance. In peer review the manuscripts are evaluated before they are published. It is a quality control system that requires that all new knowledge must be scrutinized. The rational of peer review is that it adds quality to the papers before they are published which is a must for all scholarly publishing.

 The world Peer is often defined as a person of equal standing. Peer Review can also be done following the publication. Published papers often continue to be studied and debated for a longer period and by a much wider audience.Peer review is the evaluation of creative work or performance by other people in the same field in order to maintain or enhance the quality of the work or performance in that particular field. It is a quality control system that requires all new scientific discoveries idea and implications to be scrutinized by critics and expert scientists before they become widely accepted. Peer review is impotent because it is a logical approach to scholarly activities. It is an essential component of scholarly publishing. It is also absolutely fundamental to the decision making process for publication. 

 Continuing Prof. Rahmatoolah said that there is lot criticism of peer review by the authors and readers who feel that at times the whole process is very slow, expensive, unreliable, inconsistent and potentially biased. Peer Review process varies from journal to journal and reviewers to reviewers. Some people gain and some people lose and some get which they don’t deserve. Peer Review suffers from reviewers and authors, editor’s behaviors, misconduct and inconsistency. He also talked about lack of objective and scientific criteria for reviewing, lack of contrast reviewing and lack of good subject specialists. Sending papers to wrong reviewers,changing reviewing criteria too often also affects the whole process. There is a tendency on the part of editors to enlist reviewers who are friendlier to him/her. Some reviewers may have a built in bias against highly original work and results. Some reviewers may not like some original work. At times low quality papers get published while high quality papers are rejected. Some time there is misuse by some researchers for getting position. Plagiarism, he felt, is not identified if the peer reviewers are not qualified people.Publishing quality papers is critical for all academics and researchers to find or retain a job, to get promotion besides research grants. Hence if not performed thoroughly peer review can lead to a big problem or tragedy, he remarked.

 Offering a solution to these problems, he suggested going for objective evaluation from subjective approach. Developing objective criteria, preventing flaws in evaluation, learning critical creative thinking which many of us lack. We must improve our mentality about judgment. Be supportive of good research by young scholars, maintain consistency in reviewing and help the scholar’s community to maintain healthy approach to knowledge production and dissemination. We must go for originality, novelty, quality, usefulness and effectiveness while reviewing papers. Speaking about the approach to peer review Prof. Rahmatullah said that classic approach consists of evaluating a paper on the basis of a check list i.e. title, abstract, key words, problems statement, literature review, questions or hypothesis , findings of the study, discussion and conclusions followed by relevant latest references. We must see if the paper is worth publication, it has some theoretical foundations, there is objective use of literature, and there is originality with proper research design, its usefulness, relevance and whether it does add something to the existing knowledge. All this must be done in detail with proper evidence. It must also be considered if the topic selected is relevant to the journal, the authors have followed the instructions, message is clear and conveyed effectively. Look at the English language and grammar carefully. Find out if a pilot study was undertaken by the authors to test the methodology. After critical discussion the manuscript must provide some new knowledge. Look at the integrity and qualitative criterion and the possible chances of its citation thereby affecting the Impact Factor of the journal. Look if you have published similar papers earlier and what is going to be the reactions of the readers and whether it is in line with the aims and objectives of the journal?

 He concluded his presentation by saying that knowledge is produced through research. Knowledge is accessible through scholarly paper; it links past, present and future knowledge. We must develop objective criteria. Scholarly papers are evaluated through the peer review process. Universities and research centers live in a competitive environment. Scholarly publishing is a must for universities to improve their ranking. Peer review is a scholarly behaviour maintaining quality in publishing research findings and like any other behaviour, it has its own problems and drawbacks. Issues concerning peer review need scholarly approach to solve them. Maintaining and enhancing the quality of papers are the main objectives of scholarly approaches and all these need to be learnt. Very few people have received formal training on how to review a paper. Hence we need to study about our job, attend workshops, and share our knowledge and experience with other colleagues. Finally we need to develop objective and rigorous criteria for comprehensive evaluation of manuscripts.

**Dr. Mohammad Reza Ghani** from ISC spoke on the citation issues and the role of journals in scholarly communication. He discussed in detail the various components of research papers and importance of citations. Citations, he said, are important for universities, researchers, in pursuit of knowledge, claims of procedures, prevention of scientific misconduct. Ideas should be possible to trace. He also referred to gaps in tracking of citations, hence one should pay attention to correct quotation of citations. Citation errors occur when authors cite it in the text and during data entry process of databases. No system exists in determining rate of clerical errors. Some other issues in citation include citation by first author, second author, Sir Name and First name which is sometimes replaced. Sometimes authors name is abbreviated. He suggested that if one uses a source cited in another source, it is important to name the original source. Sometimes there is broken data in text or there are two or more works by same author the same year. In such cases cite A & B with author’s name instead of IBID and the cited author is repeated.

**Dr. Sholeh Arastoopour** also from ISC talked about Review papers- a possible solution for information overload. Review papers, she said, scrutinize current knowledge. She also highlighted the benefits of review papers which helps researchers keep up-to-date, show on what topics the colleagues are working on. He also talked about narrative and systematic reviews and differences between both these reviews. Selection of topic and author is very important. Reviewer must have creativity and must be an expert on that particular topic.

## Ethical issues in Scientific Publishing

**Ms. Sarah Masoumi** from Iranian Journal of Medical Sciences made a presentation on behalf of **Dr. Behrooz Astaneh** on ethical issues in scientific publishing. This presentation covered in detail topics like ethical misconduct, author disputes, conflict of interest, redundant publication, duplicate submission, fraud, plagiarism, data fabrication and data falsification. Reasons for ethical misconduct could be intentional or due to lack of knowledge. Many researchers do not know what is considered as scientific misconduct. Even some editorial board members are not aware of exact definitions of various misconducts. Not only that many editors do not know how to tackle misconduct. This presentation covered various examples of fraud including Woo Suk Hwang of South Korea who had claimed of stem cell cloning. Committee on Publication Ethics (COPE) it was stated is promoting integrity in research publications. COPE provides advice and resources to editors and publishers on all aspects of publication ethics.

 COPE work, it was stated, is guided by a Council and specific projects are managed by various committees. COPE was founded in 1997 and today it has over seven thousand members and it covers all disciplines. It has eighteen council members from seven countries. COPE has produced various guidelines and Flow Charts to tackle scientific misconduct. These flowcharts cover plagiarism, fabricated data, changes in authorship, Ghost, Gift and Guest authorship, conflict of interest, General suspected ethical concerns, reviewer’s misconduct and how COPE deals with complaints. COPE also publishes newsletter which is quite informative.

**Mr. Shaukat Ali Jawaid** Managing Editor of Pakistan Journal of Medical Sciences and Secretary General of EMAME also briefly addressed the participants. He pointed out after going online; there has been a manifold increase in overall submissions to Pakistan Journal of Medical Sciences in general and from Iran in particular. During the Year 2011, Pak J Med Sci received 931 manuscripts and published 307 manuscripts after peer review. During the same year the number of manuscripts received from Iran was 292 and only 78 were published after peer review. Some of these manuscripts had to be revised by the authors more than once responding to reviewer’s comments and suggestions before they were accepted. The problem which I want to highlight, he said, was the pressure from authors who are very impatient. Some of them then indulge in simultaneous submissions to other journals and also get them published without withdrawing these manuscripts from Pak J Med Sci. Hence, much against our wishes, we had to take some unpleasent action black listing some of these authors who indulged in scientific misconduct who were also reported to their institution heads. We always advise the authors to publish their manuscripts in the journals from their own country but also offer them help and assistance in publications but scientific misconduct cannot be tolerated, he added.

 During the discussion it was pointed out that efforts should be made to collect all relevant literature, enlist co-authors if necessary, and enter citations into an electronic database. Readers, it was stated, do look for new ideas and specific information which clarifies these ideas. Systemic reviews are considered more useful than narrative reviews. Narrative Reviews if done by a distinguished author makes a difference.

## Scientific misconduct

 The next session was devoted to scientific misconduct. **Dr. Hamid R. Jamali** was the first speaker who talked about plagiarism and journal publishing. Plagiarism, he said, is defined as the use of someone else’s work or ideas without attribution. It can also be in the shape of paraphrasing and summarizing. It could be in the shape of copying without proper referencing, misattribution, missing in text citation,fabricating references, paraphrasing without rewriting, misinterpretation and self plagiarism. It is important to avoid plagiarism hence one should be very cautious about it. For research we rely on past work and one should write it in one’s own words.

 Speaking about types of plagiarism, he mentioned clear plagiarism and minor plagiarism. It could be an intention to deceive. Punishment for students, young researchers, he opined, should be modest as compared to full faculty members who should get severe punishment for plagiarism. Language barriers are yet another factor why people indulge in plagiarism. In order to detect plagiarism, editors should screen all manuscripts on submission before they are accepted for peer review. Every journal should have a written policy on dealing with plagiarism. Punishment decisions should be based on type of plagiarism. One should be fair and consistent and editorial policy of the journal should give full details. Authors involved in plagiarism can be banned from further submissions for a particular period. These authors can also be reported to their respective Ministry of Health, Universities and institution heads. He also suggested that every country should have a national body to monitor scientific integrity and efforts should be made to discourage plagiarism.

**Dr. Hamid Alizadeh** presented a review of plagiarism detection soft ware’s and services. Plagiarism it was stated is a worldwide problem. He also referred to the role of internet and other Information and Communication Technology (ICT) tools in growth of plagiarism. Lack of research ethics was also responsible for it to some extent. Journal editors deal with these problems quite often. He described it as a social illness and called for honest policies and punishment system. Bad writing behaviour is a disease which should be detected and treated. Implementing preventive methods, it was stated, will give positive results in long term. There are numerous tools; soft ware’s now available to detect plagiarism which takes very little time.

 During the discussion it was stated that we need to create awareness and educate healthcare professionals, researchers about plagiarism. It will prevent plagiarism due to ignorance. Reasons for plagiarism in different countries may be different. Professional research ethics should be taught as a part of training to postgraduates and young researchers. To overcome weakness in English language among young researchers, they should be given courses in English language. Young medical students also need to be taught and trained in research ethics. One of the participants suggested that the journals should put the manuscripts received online for some time and if there is no complaint only then process it further for publication. Peer Review as well as Editing a journal is a time consuming job. Institution heads like Vice Chancellors or Deans are already contributing in many ways hence it should not be compulsory for them to have more published papers for further promotions.

**Figure:**
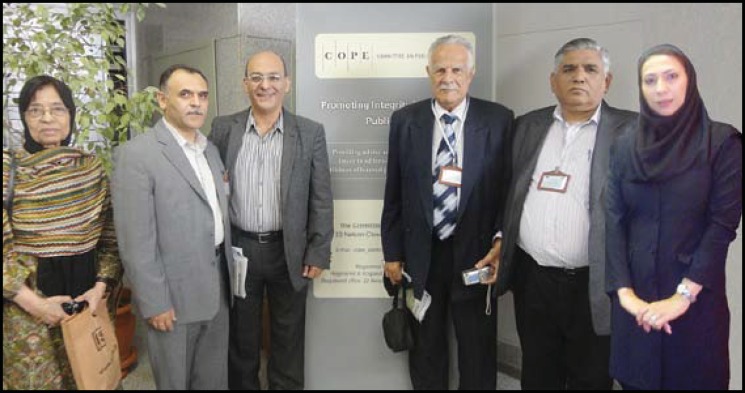
Photograph taken during the ICS conference at Shiraz shows from (L to R) Dr. Fatema Jawad from Pakistan, Dr. Mohammad Reza Ghani from ICS, Dr. Behrooz Astaneh from Iran, Dr. Ahmad Saeed Al-Morsi from Egypt, Mr. Shaukat Ali Jawaid from Pakistan & Ms. Sara Masoumi from Iran.

## Islamic World Science Citation Center (ISC)

 On second day of the conference the session was devoted to inform and update the participants about the services offered and functioning of Islamic World Science Citation Center (ISC). **Prof. Farideh Osareh** from Shahid Chamran University of Ahvaz talked about research journals and citation studies. Science, she said, is an international activity. Quality, visibility and impact of research journals are important. She mentioned about different criteria’s to improve quality of research journals. Citation analysis, she pointed out, was a research method for evaluating the influence and productivity of scientific and technical communication. Journals are a major channel of communication among scholars. Journals publishing represent heart of scientific and technical communication. We must find out what can be done to improve the quality, visibility and Impact Factor of journals from developing countries. Without internalization, science, she opined, cannot flourish. One cannot guarantee standards. Participation of developing countries in science is vital. Tropical diseases are more prevalent in developing countries. There is a lack of proper scholarly communication. If no appropriate steps are taken, science in developing countries will be isolated. No one will read and criticize them what they are doing. New ideas will reach them slowly. If a journal has more citations, it means better quality. Multinational collaboration improves visibility. Multinational distribution of editorial team, multinational authors, users, advisors and international collaboration all are very helpful in improving the standard, quality and visibility of scientific journals.

**Ms. Mansooreh Serati** from ISC pointed out that ISC Journal Citation Report (JCR) is an important tool used in ranking of journals. ISC prepares JCR in Persian, Arabic & English. ISC website www.isc.gov.ir gives all relevant details.

**Ms. Forough Rahimi** also from ISC made a presentation on ISC’s Science Citation Index. She pointed out that ISI Thompson Reuter was established in 1960 while Scoups was introduced in 2004. ISC made its debut in 2008 for evaluating research performance of Islamic countries. It is helpful to Islamic countries. Now many other countries outside the Islamic World have also shown interest in joining ISC which will make it an international database.

**Dr. Ali Gazni** from ISC highlighted ISC’s contribution reports while **Dr. Hamid Alizadeh** described in detail the ISC Scientific Journal Submission System. Provision of XML files of the manuscripts, he stated, offers ease in processing of articles. During the discussion it was pointed out that those journals which cannot generate XML files can send the soft copy or printed copy of their journals and they will be processed further.

## Concluding Session

 In the concluding session there was interactive discussion on most of the points discussed during the two day meeting and the issues raised. In the end **Mr. Shaukat Ali Jawaid** from Pakistan summarized the suggestions, recommendations of the conference based on the discussions and presentations. This was as under:

1. Participants of this First International meeting of Research Journal Editors appreciates & congratulates ISC for this initiative of holding the first conference of Research Journal Editors from Islamic countries. Such meetings should be organized on regular basis in different countries.
2. ISC should be provided with hard or soft copies of all manuscripts by Research Journal Editors on regular basis to strengthen this database which will be of immense help and benefit for researchers.
3. ISC should broaden its base by having focal persons in all the Islamic countries to facilitate it in achieving its objectives.
4. ISC should co-operate with Eastern Mediterranean Association of Medical Editors (EMAME) and Prof. Farhad Handjani President-elect of EMAME who is on the faculty of Shiraz University of Medical Sciences can be contacted to work out the details of this cooperation.
5. Research Journals should have an author friendly policy to help, guide and assist them in improving the quality of their manuscripts.
6. The role of Professional Writers should be recognized and to ensure transparency their help and assistance in preparation of the manuscripts should be acknowledged.
7. All institutions should play their role in educating and creating awareness about plagiarism and its consequences for the authors.
8. Research Journal Editors should improve their communication with authors and keep them informed about developments regarding their manuscripts submitted for publication.
9. Workshops, Hands on Training should be organized on Medical Writing, Medical Editing and Peer Review to promote the art of scientific publishing in the Islamic World. It should be done by research journals, institutions including universities as well as professional specialty organizations. Authors should also be encouraged to use ISC database.
10. Institutional Review Board, Ethics Committee approval should be made mandatory before accepting manuscripts for publication. It will help in checking and minimizing the chances of academic fraud and scientific misconduct.
11. Students and Young researchers should be trained in checking of references, citations by the research journals and they should be paid some honorarium.
12. All participants of this meeting should report about this conference and its highlights in their respective publications and keep the ISC offices informed of what they have done or plan to do to promote the aims and objectives of ISC.
13. Speakers from ISC should be invited to various conferences organized by different countries. An invitation will be extended to ISC to participate and make a presentation on this important database in the forthcoming EMAME Congress scheduled to be held in 2013 so that medical editors from this region could be informed about its functioning.

The participants thanked the ISC management for its exceptional hospitality. The delegates were also provided an opportunity to visit the tombs of famous poets Hafiz and Saadi Shirazi in the evening on the first day of the conference.

